# The Effects of Fat Body Tyramine Level on Gustatory Responsiveness of Honeybees (*Apis mellifera*) Differ between Behavioral Castes

**DOI:** 10.3389/fnsys.2017.00055

**Published:** 2017-08-08

**Authors:** Ricarda Scheiner, Brian V. Entler, Andrew B. Barron, Christina Scholl, Markus Thamm

**Affiliations:** ^1^Behavioral Physiology and Sociobiology, Biocenter, University of Würzburg Würzburg, Germany; ^2^Department of Biological Sciences, Macquarie University Sydney, NSW, Australia

**Keywords:** biogenic amines, division of labor, nurse bee, forager, PER, octopamine, insect, behavior

## Abstract

Division of labor is a hallmark of social insects. In the honeybee (*Apis mellifera*) each sterile female worker performs a series of social tasks. The most drastic changes in behavior occur when a nurse bee, who takes care of the brood and the queen in the hive, transitions to foraging behavior. Foragers provision the colony with pollen, nectar or water. Nurse bees and foragers differ in numerous behaviors, including responsiveness to gustatory stimuli. Differences in gustatory responsiveness, in turn, might be involved in regulating division of labor through differential sensory response thresholds. Biogenic amines are important modulators of behavior. Tyramine and octopamine have been shown to increase gustatory responsiveness in honeybees when injected into the thorax, thereby possibly triggering social organization. So far, most of the experiments investigating the role of amines on gustatory responsiveness have focused on the brain. The potential role of the fat body in regulating sensory responsiveness and division of labor has large been neglected. We here investigated the role of the fat body in modulating gustatory responsiveness through tyramine signaling in different social roles of honeybees. We quantified levels of tyramine, tyramine receptor gene expression and the effect of elevating fat body tyramine titers on gustatory responsiveness in both nurse bees and foragers. Our data suggest that elevating the tyramine titer in the fat body pharmacologically increases gustatory responsiveness in foragers, but not in nurse bees. This differential effect of tyramine on gustatory responsiveness correlates with a higher natural gustatory responsiveness of foragers, with a higher tyramine receptor (*Amtar1*) mRNA expression in fat bodies of foragers and with lower baseline tyramine titers in fat bodies of foragers compared to those of nurse bees. We suggest that differential tyramine signaling in the fat body has an important role in the plasticity of division of labor through changing gustatory responsiveness.

## Introduction

Honeybee colonies display a complex yet highly plastic social organization. Each bee performs a series of social tasks (for review see Johnson, 2010). The most important changes in individual behavior occur when nurse bees switch to foraging tasks. While nurse bees stay inside the hive and provide the young larvae with food, foragers leave the hive daily to forage for nutrients, i.e., pollen and nectar (Johnson, 2010). Nurse bees are normally between 1 week and 2 weeks of age. Foragers leave the hive at roughly 3 weeks of age. Although division of labor clearly depends on the age of the individual, age *per se* cannot account for the behavioral transitions. Because of the great plasticity in division of labor, the honeybee offers the unique opportunity to dissociate age from social role, which is difficult in most other species. Thus it was shown that under certain conditions nurse bees and foragers can be identical in age (Behrends et al., 2007; Scheiner and Amdam, 2009) and that most changes in physiology and gene expression of differently aged bee groups are associated with the behavior of the bee rather than with her age (Toth and Robinson, 2005; Alaux et al., 2009).

A recent hypothesis (Ament et al., 2010) suggests that nutrition-related signaling cascades are involved in regulating and controlling division of labor in a honeybee colony. In support of this theory, foragers differ from nurse bees in their nutrition-related gustatory response thresholds (Thamm and Scheiner, 2014). Further, nurse bees and foragers differ in the amounts of lipids they store in the fat body, with nurse bees losing their lipid stores during the transition to foraging behavior (Toth and Robinson, 2005). Reducing lipid stores pharmacologically by treating bees with a fatty acid synthesis inhibitor similarly induced precocious foraging behavior (Toth et al., 2005). When colonies were deprived of food, bees began foraging earlier than did bees from well-fed colonies (Schulz et al., 1998). In addition, it was shown that the fat body has an important function in honeybee metabolism (Nilsen et al., 2011; Wang et al., 2012), which differs hugely between nurse bees and foragers. These experiments suggest an important role for the honeybee fat body in regulating and modulating honeybee division of labor, possibly through modulation of sensory response thresholds. However, investigations on the functions of the fat body in controlling honeybee behavior are rare. A direct effect of the fat body on behavior was demonstrated when the gene encoding the egg yolk precursor vitellogenin was downregulated in the fat body of honeybees. Reduction in gene expression significantly enhanced responsiveness to gustatory stimuli (Amdam et al., 2006).

We here investigated the role of fat body tyramine signaling in regulating and modulating gustatory responsiveness in nurse bees and honeybee foragers. Tyramine is the metabolic precursor of octopamine. While the latter has been studied in detail, very little is known about tyramine, although two honeybee tyramine receptors have been cloned and characterized (Blenau et al., 2000; Reim et al., 2017). The tyramine receptor gene *Amtar1* (also known as *Amtyr1*) is a candidate gene in a quantitative trait locus that correlates with different aspects of foraging-related behaviors in two honeybee strains that differ in their gustatory responsiveness (Hunt et al., 2007). Furthermore, tyramine injections into the thorax can increase gustatory responsiveness (Scheiner et al., 2017). We address the relationships between tyramine titer and tyramine receptor gene expression in the fat body and gustatory responsiveness in nurse bees and foragers. Additionally, we studied how injection of tyramine into the abdomen affected tyramine and octopamine titers in the brain and fat body and whether it modulated gustatory responsiveness. Intriguingly, tyramine application to the abdomen has different effects on gustatory responsiveness in nurse bees and foragers.

## Materials and Methods

### Bees

Nurse bees and foragers were randomly obtained from typical honeybee colonies comprising approximately 40,000 honeybees. Honeybees (*Apis mellifera ligustica*) used for determining biogenic amine titers were sampled from hives maintained at Macquarie University Sydney, Sydney, NSW, Australia. Honeybees (*Apis mellifera carnica*) for behavioral analyses, behavioral pharmacology and gene expression studies were kept at the departmental apiary of the University of Würzburg.

Nurse bees were collected from frames containing open brood cells. Only bees poking their heads into an open brood cell for at least 15 s were regarded as nurse bees. Foragers were collected when returning to their colonies. For behavioral pharmacology of nurse bees, frames with capped broods were kept in an incubator maintained at 34°C and 65% humidity until the bees emerged. Newly emerged bees received paint-marks on their thoraces and were restored to the colony. After 1 week, when most of these bees performed nursing tasks, bees were individually retrieved from the colony (Thamm and Scheiner, 2014).

### Gustatory Responsiveness

For testing gustatory responsiveness, each bee was immobilized on ice and subsequently mounted in a small holder with antennae and mouth parts protruding. Tests commenced 60 min after mounting (Scheiner et al., 2013). During this time, the bee rested in a humidified chamber. For determining gustatory responsiveness, each bee was sequentially stimulated by application of a series of sucrose concentrations (0%; 0.1%; 0.3%; 1%; 3%; 10%; and 30% w/v) to her antennae (for details see Scheiner et al., 2013). The sum of proboscis extension responses to stimulations with the seven different sucrose concentrations constitutes the gustatory response score (GRS) of a bee, which is a measure of its gustatory responsiveness (Scheiner et al., 2003, 2013; Scheiner, 2004; Behrends and Scheiner, 2010; Scheiner and Arnold, 2010; Thamm and Scheiner, 2014). For determining GRS of nurse bees and foragers without tyramine treatment, 106 nurse bees and 121 foragers were tested. These bees were also used to study effects of different concentrations of tyramine on gustatory responsiveness.

### Behavioral Pharmacology

Different tyramine concentrations (10^−2^ mol/l and 10^−3^ mol/l) were dissolved in phosphate-buffered saline solution (PBS: 140 mM NaCl, 2.6 mM KCl, 8.1 mM Na_2_HPO_4_ and 1.5 mM KH_2_PO_4_; pH 7.4). Bees were either injected with 2 μl of a tyramine solution or with 2 μl of the PBS solution (“control”) into the abdomen. For this, each bee was punctured between the fourth and fifth tergites and injected with a Hamilton syringe (10-μl syringe, Hamilton Bonaduz AG, Switzerland). To evaluate the effect of tyramine, changes in GRS 30 min after application of tyramine compared to GRS prior to treatment were calculated and compared between groups.

### Quantification of mRNA

Individual fat body tissues of nurse bees and foragers that had not been used in behavioral tests were homogenized in 750 μL of *Isol-RNA lysis reagent* (5PRIME, Hilden, Germany). Afterwards, 150 μL of chloroform were added. After phase separation, the aqueous phase was transferred into 900 μL ethanol (75%). Subsequently, the *peqGOLD Total RNA Kit* (Peqlab, Erlangen, Germany) was employed to purify RNA following the standard protocol including DNase I digestion step. From each bee, we transcribed 500 μg of total fat body RNA using *QuantiTect*^®^ Reverse Transcription Kit (Qiagen, Hilden, Germany). Of each cDNA, we amplified 5 μL in triplicates in a quantitative real time PCR on a Rotor-Gene Q (Qiagen, Hilden, Germany) using the following protocol: 1 min at 60°C, 5 min at 95°C and 45 cycles consisting of 20 s at 95°C followed by 1 min at 60°C each. One reaction (25 μL) contained each primer (0.25 μM), TaqMan^©^ probes (0.1 μM) and Rotor-Gene Multiplex PCR Master Mix (Qiagen, Hilden, Germany). Sequences of primers and Taqman probes are given in Table 1. Relative expression to transcript of Am*EF1α* (Reim et al., 2013) with the ΔΔCt method was determined using Rotor Gene Q software (Qiagen, Chatsworth, CA, USA). The same bees were investigated with respect to mRNA expression of the three genes. Nevertheless, direct comparisons between mRNA expression of different genes within individuals were inappropriate due to possible differences in primer and probe efficiencies.

**Table 1:** DNA-Oligos used for quantitative real time PCR.

Gene	Oligo name	Oligo sequence
*AmEF1α*	AmEF1α_qF	GAACATTTCTGTGAAAGAGTTGAGGC
	AmEF1α_qR	TTTAAAGGTGACACTCTTAATGACGC
	AmEF1α_TM	6FAM-ACCGAGGAGAATCCGAAGAGCATCAA-BBQ
*Amtar1*	AmTar1_F	AGCCGACCGAGGTCACGATAG
	AmTar1_R	CCCATTATCACGCCCAATGTCC
	AmTar1_TM	YAK-AACGAGATCCTCTGCCTCTCCTCGATGAA-BBQ
*Amtar2*	AmTar2_F	GTTACTAATTGTTTCGTGTCCAGCTT
	AmTar2_R	GCAGTACAGAGAAGAATGTCGAGG
	AmTar2_TM	YAK-AGGTACCACCTGTGAGCTGTAACAGCA-BBQ
*AmoctαR1*	AmOa1_F	GCAGGAGGAACAGCTGCGAG
	AmOa1_R	GCCGCCTTCGTCTCCATTCG
	AmOa1_TM	YAK-TCCCCATCTTCATCACCCTTGGCTTCTCC-BBQ

### Quantification of Tyramine and Octopamine Titers

To measure natural levels of tyramine and octopamine in the fat bodies of nurse bees and foragers, bee samples were collected, and the abdomens dissected under PBS to remove fat bodies. Dissections were performed as quickly as possible, and fat body samples were then stored at −80°C. To determine whether injection with tyramine into the abdomen would lead to elevated tyramine or octopamine titers in the brain or fat body, individuals were treated by abdominal injection as described above. Thirty minutes after treatment, the head of each bee was quickly cut from the body at the neck and immediately flash frozen in liquid nitrogen. The abdomen was stored briefly on dry ice, and then dissected under PBS to remove the fat body. Heads were lyophilized at −65°C and 320 mTorr for 50 min to remove some water content. Brains were dissected from head capsules over dry ice while frozen. Dissected brains were then stored at −80°C until further processing.

For extraction of biogenic amines from brains or fat body samples, tissue was first centrifuged at 15,000 *g* for 2 min at 4°C to induce mechanical disruption of tissue. Samples were then homogenized by sonication in 100 μL of 0.2 M perchloric acid containing 10 pg/μL dihydroxybenzylamine (DHBA). Homogenized samples were incubated on ice in darkness for 20 min, before centrifugation at 15,000 *g* for 15 min to pellet cell fragments. The supernatant was collected, and 10 μL of the supernatant of each sample were analyzed with high-pressure liquid chromatography (HPLC). Content of biogenic amines in the extractant from tissue samples was quantified using an Agilent 1200 Series HPLC system (Agilent Technologies, Santa Clara, CA, USA) with an ESA Coulechem III electrochemical detector connected to an ESA 5011A dual electrode analytical cell (ESA, Chelmsford, MA, USA). Samples were separated across a 100 mm Thermo Fisher Scientific Hypersil 5 μm octadecylsilane packaged column (Thermo Fisher Scientific, Waltham, MA, USA). Biogenic amine amounts were quantified relative to known amounts of biogenic amines (Søvik et al., 2013; Scheiner et al., 2014b). The bees whose natural amine titers were measured were different from those treated with tyramine or PBS.

### Statistics

Biogenic amine brain titers, GRSs and changes in GRSs were compared between different groups using two-tailed Mann Whitney *U* tests, since data were not distributed normally. For comparing more than two groups, we employed Kruskal-Wallis *H* tests followed by Dunn’s *post hoc* tests. Due to the high individual variability of these data, we show individual data points with super-imposed medians for biogenic amine data. Many GRS values were “0” or “1”. For clarification, GRS data are therefore represented by medians and upper and lower quartiles. GRS before and after treatment were compared within each group using two-tailed Wilcoxon tests. Relative amine receptor gene expression was compared between groups using *T*-tests, since these data were distributed normally. All tests were two-tailed. Comparisons were performed using GraphPad Prism (GraphPad Software, Inc., La Jolla, CA, USA).

## Results

### Gustatory Responsiveness of Nurse Bees and Foragers

**Figure 1:**
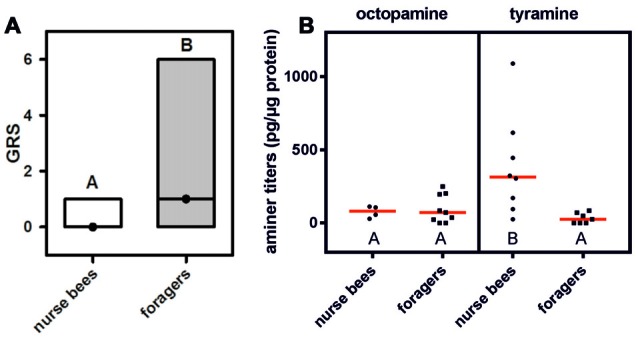
Gustatory responsiveness and tyramine titers in the fat body differ between nurse bees and foragers. **(A)** Gustatory response scores (GRSs) of foragers and nurse bees. Medians (dots) and upper and lower quartiles (lines) are displayed. Groups with different letters differ significantly (*P* at least <0.05, Mann Whitney *U* test). **(B)** Octopamine and tyramine titers in the fat bodies of nurse bees and foragers. Figures display individual data points and medians (red lines). Groups with different letters differ significantly (*P* at least <0.05, Mann Whitney *U* test).

We first wanted to demonstrate that in our honeybee population we could replicate the finding that foragers have a higher gustatory responsiveness than nurse bees do (Thamm and Scheiner, 2014). We therefore quantified gustatory responsiveness in both social roles. Foragers displayed significantly higher GRSs than did nurse bees (Figure 1A: *Z* = 4.88, *n*_nurse bees_ = 106, *n*_foragers_ = 121, *P* < 0.001, Mann Whitney *U* test), demonstrating a higher general responsiveness to all sucrose stimuli offered (Scheiner et al., 2014a).

### Amine Titers in the Fat Body of Nurse Bees and Foragers

We hypothesized that behavioral differences of nurse bees and foragers could coincide with different amounts of tyramine in their fat bodies. Tyramine is the metabolic precursor of octopamine (Roeder, 2005), and both amines can have similar effects on behavior (Scheiner et al., 2002, 2006). We therefore decided to quantify both amines in the fat bodies of nurse bees and foragers. Octopamine was present in very low amounts in the fat bodies of nurse bees and foragers, and its level did not differ between the two social roles (Figure 1B; *Z* = 0.88, *n*_nurse bees_ = 4, *n*_foragers_ = 9, *P* > 0.05). Intriguingly, foragers displayed significantly lower tyramine titers in their fat bodies than nurse bees did (Figure 1B; *Z* = 2.90, *n*_nurse bees_ = 8, *n*_foragers_ = 7, *P* < 0.01). These data suggest differential tyramine signaling in nurse bees and foragers, while octopamine titers did not differ between social roles.

### Expression of Tyramine and Octopamine Receptors in the Fat Bodies of Nurse Bees and Foragers

This experiment was aimed at finding out whether expression of tyramine receptors in the fat body correlates with social role. We therefore quantified the mRNA of both honeybee tyramine receptor genes, *Amtar1* and *Amtar2*, in the fat bodies of nurse bees and foragers. In addition, we measured expression of the octopamine receptor *AmoctαR1*, because mRNA expression of this receptor differs hugely between the brains of nurse bees and those of foragers (Reim and Scheiner, 2014), and tyramine can bind to octopamine receptors at high concentrations (Blenau et al., 2000; Reim et al., 2017).

**Figure 2:**
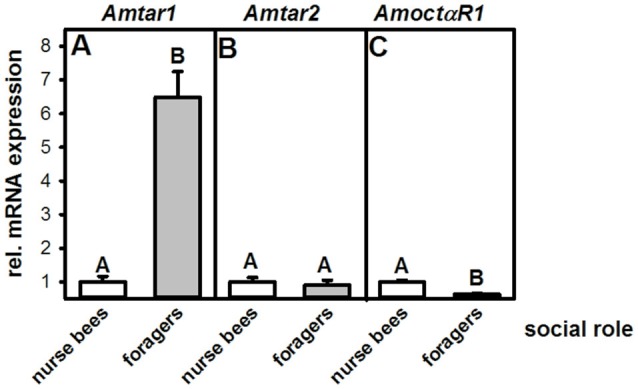
Relative mRNA expression of octopamine and tyramine receptors in the fat bodies of nurse bees and foragers. **(A)** *Amtar1*. **(B)** *Amtar2*. **(C)** *AmoctαR1*. Foragers have a significantly higher expression of the tyramine receptor *Amtar1* gene than nurse bees. The figure shows means and standard errors. Groups with different letters differ significantly (*P* at least <0.05, *T* test).

The mRNA expression of the tyramine receptor *Amtar1* was significantly increased in foragers compared to nurse bees (Figure 2A; *n*_nurse bees_ = 10, *n*_foragers_ = 8, *T* = 6.95, *P* < 0.001, *T*-test). Expression of *Amtar2* mRNA, in contrast, did not differ between fat bodies of foragers and nurse bees (Figure 2B; *n*_nurse bees_ = 7, *n*_foragers_ = 10, *T* = 0.47, *P* > 0.05). Further, foragers displayed a significantly reduced mRNA expression of the octopamine receptor *AmoctαR1* compared to nurse bees (Figure 2C; *n*_nurse bees_ = 8, *n*_foragers_ = 8, *T* = 5.91, *P* < 0.001). These data suggest that differences in the metabolism, physiology or behavior of nurse bees and foragers might be related to tyramine signaling in the fat body through *Amtar1* and possibly also through octopamine signaling through *AmoctαR1*.

### Tyramine Increases Gustatory Responsiveness in Foragers but Not in Nurse Bees

To test whether changing tyramine titers would increase gustatory responsiveness, we injected tyramine into the fat body of nurse bees and foragers. Preliminary experiments showed that injecting 1 μl of tyramine 10^−2^ mol/l into the abdomen did not affect gustatory responsiveness in honeybee foragers (*n*_control_ = 39, *n*_tyramine_ = 40, *Z* = 1.04, *P* = 0.30, Mann Whitney *U* test), although this volume and concentration can effectively increase gustatory responsiveness in foragers when injected into the thorax (Scheiner et al., 2002). We therefore tested whether injection of 2 μl of tyramine in the concentrations of 10^−2^ mol/l and 10^−3^ mol/l into the abdomen of foragers and nurse bees would affect their gustatory responsiveness.

**Figure 3:**
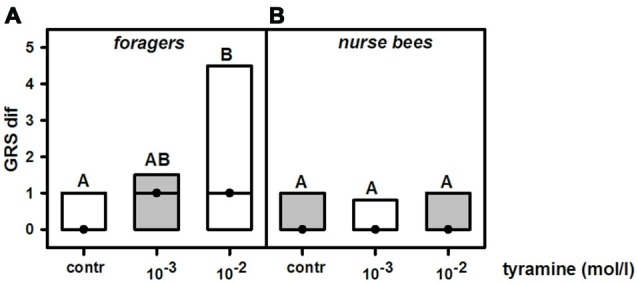
Changes in gustatory response scores (GRS dif) after tyramine treatment in foragers and nurse bees. **(A)** Foragers become significantly more responsive after treatment. **(B)** Nurse bees do not change in their gustatory responsiveness. Medians (dots) and upper and lower quartiles (lines) are displayed. Groups with different letters differ significantly (*P* at least <0.05, Mann Whitney *U* test).

Treatment with tyramine had a significant effect on gustatory responsiveness in foragers (KW = 10.01, *P* < 0.01). However, only tyramine at 10^−2^ mol/l significantly increased GRSs in this group (Figure 3A; tyramine 10^−3^ mol/l vs. control: *n*_control_ = 39, *n*_tyramine 10^−3^ mol/l_ = 41, *P* > 0.05; tyramine 10^−2^ mol/l: *n*_control_ = 39, *n*_tyramine 10^−2^ mol/l_ = 41, *P* < 0.01). In contrast to foragers, gustatory responsiveness in nurse bees was not affected by tyramine injections (Figure 3B; KW = 0.31, *P* > 0.05). Neither tyramine concentration affected GRSs in this group significantly (tyramine 10^−3^ mol/l: *n*_control_ = 35, *n*_tyramine 10^−3^ mol/l_ = 36, *P* > 0.05; tyramine 10^−2^ mol/l: *n*_control_ = 35, *n*_tyramine 10^−2^ mol/l_ = 35, *P* > 0.05). Comparison of GRS within each treatment group showed that only in foragers that were treated with tyramine in the concentrations of 10^−3^ mol/l and 10^−2^ mol/l did GRS significantly increase after treatment (foragers: tyramine 10^−3^ mol/l: *n* = 41, *Z* = 2.44, *P* < 0.05, tyramine 10^−2^ mol/l: *n* = 41, *Z* = 4.18, *P* < 0.001). Treatment of foragers with the control solution did not increase gustatory scores significantly (*n* = 39, *Z* = 1.59, *P* < 0.05). In nurse bees, neither the control group nor the two groups treated with different concentrations of tyramine changed their GRSs significantly after treatment (control: *n* = 35, *Z* = 1.16, *P* > 0.05, tyramine 10^−3^ mol/l: *n* = 36, *Z* = 1.40, *P* > 0.05, tyramine 10^−2^ mol/l: *n* = 35, *Z* = 1.58, *P* > 0.05, Wilcoxon test). These data imply that the action of tyramine on gustatory responsiveness is dependent on social role.

### Effects of Tyramine Injections on Amine Titers in the Fat Body and Brain

To test whether our differential effects of tyramine on gustatory responsiveness in foragers and nurse bees were related to biogenic amine titers, we quantified tyramine and octopamine titers in the fat bodies and in the brains of foragers and nurse bees after injections of tyramine (10^−2^ mol/l) into their abdomens. Since octopamine can have similar effects on gustatory responsiveness as tyramine (Scheiner et al., 2002; Behrends and Scheiner, 2012) and tyramine can be converted into octopamine (Roeder, 2005), we also quantified octopamine titers in the brain and abdomen. This was to ensure that only tyramine levels but not octopamine levels were elevated in the treatment groups.

**Figure 4:**
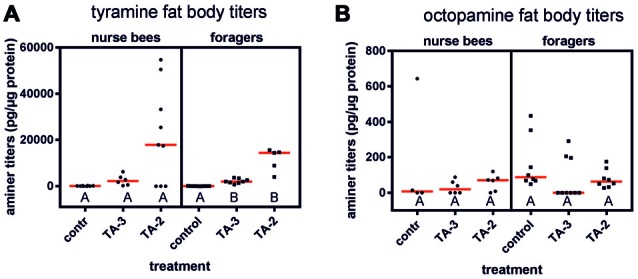
Changes in fat body amine titers after injection of tyramine into the abdomen. **(A)** Tyramine titers were not increased significantly after tyramine (TA) treatment in nurse bees but were in foragers. **(B)** Octopamine titers were not increased after tyramine treatment in nurse bees or in foragers. Medians (red line) and individual data points are displayed. Groups with different letters differ significantly (*P* at least <0.05, Kruskal Wallis *H* test).

Tyramine injections into the abdomen significantly increased tyramine titers in the abdomen of foragers (Figure 4A; KW = 18.34, *P* < 0.001). Both tyramine concentrations led to a significant increase in tyramine titers (tyramine 10^−3^ mol/l vs. control: *n*_control_ = 9, *n*_tyramine 10^−3^ mol/l_ = 8, *P* < 0.05; tyramine 10^−2^ mol/l: *n*_control_ = 9, *n*_tyramine 10^−2^ mol/l_ = 5, *P* < 0.001). However, the higher tyramine concentration elevated tyramine levels in the fat body more strongly. In contrast, tyramine injections had no effect on octopamine titer in the fat bodies of foragers (Figure 4B; KW = 4.00, *P* > 0.05, *n*_control_ = 8, *n*_tyramine 10^−3^ mol/l_ = 9, *n*_tyramine 10^−2^ mol/l_ = 8, Kruskal-Wallis *H* test).

In contrast to foragers, injection of tyramine into abdomens of nurse bees did not result in increased tyramine titers in their fat bodies, although a similar trend was observable as was demonstrated in foragers (Figure 4A; KW = 5.41, *n*_control_ = 8, *n*_tyramine 10^−3^ mol/l_ = 6, *n*_tyramine 10^−2^ mol/l_ = 9, *P* > 0.05). Octopamine levels were also not elevated in the fat body of nurse bees after injections of tyramine into the abdomen (Figure 4B; KW = 1.40, *n*_control_ = 4, *n*_tyramine 10^−3^ mol/l_ = 6, *n*_tyramine 10^−2^ mol/l_ = 6, *P* > 0.05).

Since the behavioral effects observed in foragers after tyramine injection into the abdomen might have been induced by an increased tyramine titer in the brain, we also quantified tyramine levels in the brains of nurse bees and foragers after injection of tyramine into the abdomen. We here only investigated the effect of the higher tyramine concentration (10^−2^ mol/l), since only this concentration had a significant effect on behavior.

**Figure 5:**
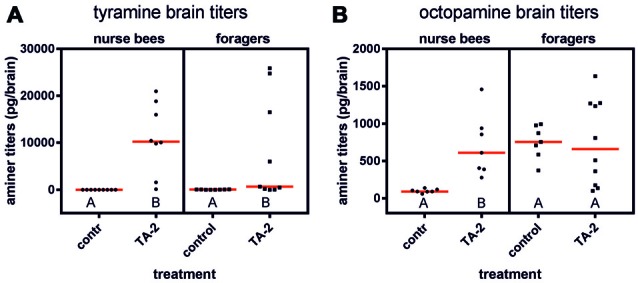
Changes in brain amine titers after injection of tyramine into the abdomen. **(A)** Tyramine titers were increased significantly after tyramine (TA) treatment in nurse bees and in foragers. **(B)** Octopamine titers were significantly increased after tyramine treatment in nurse bees but not in foragers. Medians (red line) and individual data points are displayed. Groups with different letters differ significantly (*P* at least <0.05, Mann Whitney *U* test).

After injection of 2 μl of tyramine (10^−2^ mol/l) into the abdomen, the tyramine titer was significantly elevated in the brains of forager bees (Figure 5A; *n*_control_ = 8, *n*_tyramine_ = 9, *Z* = 2.37, *P* < 0.05, Mann Whitney *U* test). The octopamine brain titer was not affected by tyramine injection (Figure 5B; *n*_control_ = 7, *n*_tyramine_ = 10, *Z* = 0.20, *P* > 0.05). In contrast to foragers, nurse bees displayed both increased tyramine and octopamine titers in their brains (tyramine: Figure 5A; *n*_control_ = 9, *n*_tyramine_ = 8, *Z* = 3.75, *P* < 0.001; octopamine: Figure 5B; *n*_control_ = 7, *n*_tyramine_ = 7, *Z* = 3.13, *P* < 0.01).

## Discussion

Our data show that foragers were significantly more responsive to sucrose than were nurse bees, confirming the link between social organization and nutrition in a honeybee colony. The higher gustatory responsiveness observed in foragers generally leads to better associative learning performance (Scheiner et al., 1999, 2001a,b, 2003) and correlates with higher visual responsiveness (Erber et al., 2006) and with a higher responsiveness to odors (Scheiner et al., 2004). Whether it is causally related to foraging tasks has yet to be shown.

The differences in gustatory responsiveness of nurse bees and foragers correlated with differences in tyramine physiology. Foragers had higher expression of the tyramine receptor gene *Amtar1* in their fat bodies than nurse bees, while displaying lower tyramine titers. Pharmacological activation of tyramine receptors increased gustatory responsiveness in foragers even further, while no significant effect was observed in nurse bees. Although some of the specifics of the modes of tyramine action presently remain unclear, it seems that tyramine metabolism in the periphery differs between individuals performing different tasks and could thus contribute to the behavioral differences between behavioral states.

The fat body has an important function in honeybee metabolism (Nilsen et al., 2011; Wang et al., 2012), which differs hugely between nurse bees and foragers. Nevertheless, the function of this organ in regulating social organization has hardly been investigated. Our data suggest that the fat body could mediate division of labor through modulating nutrition-related sensory response thresholds via aminergic signaling cascades. The tyramine receptor AmTAR1 is particularly interesting in this respect, since its mRNA expression is more than six-fold elevated in foragers compared to nurse bees. The higher expression of this tyramine receptor, which decreases intracellular cAMP levels upon activation (Blenau et al., 2000; Reim et al., 2017), might be causally related to an increased gustatory responsiveness, as observed in foragers. Future experiments in which the receptor expression is reduced in young bees, for example by RNA interference in the fat body (Amdam et al., 2006; Nilsen et al., 2011; Wang et al., 2012), should lead to a reduced gustatory responsiveness as observed in nurse bees and possibly to a delayed onset of foraging behavior. Reducing tyramine receptor expression in the fat bodies of foragers might even induce nursing behavior. In addition, foragers displayed a significantly lower expression of the octopamine receptor gene *AmoctαR1*. Intriguingly, the lower fat body *AmoctαR1* expression in foragers compared with nurse bees contrasts with the brain, where foragers have higher octopamine titers (Schulz et al., 2002) and higher *AmoctαR1* mRNA expression (Reim and Scheiner, 2014). Because both tyramine and octopamine can modulate gustatory responsiveness in the same direction, it will be interesting in the future to separate the functions of the different types of octopamine and tyramine receptors in the fat body based on sensory responsiveness and other behaviors.

Our data reveal that injections of tyramine into the abdomen of honeybees are an effective method of increasing tyramine titers in the abdomen of honeybee foragers, but not of nurse bees. In the case of foragers, this method also increases tyramine brain levels, while in nurse bees, both tyramine and octopamine brain titers were increased. These findings are important for behavioral pharmacological experiments with different age groups or behavioral groups in honeybees. Furthermore, it is interesting that nurse bees naturally have lower tyramine brain levels compared to foragers (Reim et al., 2017), which contrasts with their significantly higher tyramine titers in their fat bodies. These findings suggest that nursing behavior and foraging behavior coincide with differential tyramine signaling.

The fact that tyramine acted on gustatory responsiveness in foragers, but not in nurse bees, is challenging. Our data suggest that most likely these differences in behavioral response might have been caused by mechanisms outside the brain through differences in tyraminergic signaling in the periphery, including the fat body. Foragers showed a significantly higher *Amtar1* mRNA expression than did nurse bees in their fat bodies. Coinciding with this, foragers had a significantly lower tyramine titer in their fat bodies compared to nurse bees. An elevated tyramine titer in the abdomen could therefore effectively modulate behavior through activation of abundant tyramine receptors in foragers. It is unlikely that the effects on behavior were induced by conversion of the injected tyramine into octopamine in the fat bodies, because octopamine titers of foragers were not elevated after tyramine injection. However, we cannot exclude the possibility that the injected tyramine bound to octopamine receptors in fat bodies (Grohmann et al., 2003; Balfanz et al., 2014). In particular, nurse bees had a high expression of the octopamine receptor gene *AmoctαR1* in their fat bodies. If the respective receptor acted in an opposite way compared to the tyramine receptor AmTAR1, the activated receptor might have inhibited the behavioral effects of tyramine on gustatory responsiveness. Since nurse bees showed very low mRNA expression of *Amtar1* coinciding with high natural titer of tyramine in their fat bodies, further elevating tyramine titers in fat bodies might therefore have had little effect on behavior of nurse bees, when controlled by tyraminergic fat body regulatory mechanisms. Why nurse bees have high tyramine titers is not quite clear. Since they consume large amounts of pollen to produce brood food, they also consume large amounts of tyrosine, which is frequently present in bee-collected plant pollen (Szczêsna, 2006). This amino acid can be converted into tyramine by decarboxylation (Roeder, 2005). Also, tyramine itself has been shown to be present in many plants (Smith, 1977). It might therefore be present in the pollen grains of the plants as well. This, however, has not been investigated to the best of our knowledge.

Our results suggest that tyramine can have a decisive function in regulating division of labor though modulating gustatory responsiveness. They further imply that the fat body of honeybees may have a much more important role in controlling behavior including social organization than believed hitherto. These are important aspects for the function of this organ, which has mainly been placed in the context of metabolism. The new link between fat body tyramine signaling, gustatory responsiveness and division of labor between nurse bees and foragers strengthens the hypothesis proposed by Ament et al. (2010) that nutrition-related mechanisms control social organization in a honeybee colony.

## Author Contributions

RS designed the experiments, supervised part of the experiments, analyzed data and wrote the first draft of manuscript. BVE acquired and analyzed data and contributed to the manuscript. ABB designed the experiments, supervised part of the experiments, analyzed data and contributed to the manuscript. CS supervised part of the experiments and contributed to the manuscript. MT performed part of the experiments, analyzed data and contributed to the manuscript.

## Conflict of Interest Statement

The authors declare that the research was conducted in the absence of any commercial or financial relationships that could be construed as a potential conflict of interest.

## References

[B1] Alaux C. Le Conte Y. Adams H. A. Rodriguez-Zas S. Grozinger C. M. Sinha S.. (2009). Regulation of brain gene expression in honey bees by brood pheromone. Genes Brain Behav. 8, 309–319. 10.1111/j.1601-183X.2009.00480.X 19220482

[B2] Amdam G. V. Norberg K. Page R. E. Jr. Erber J. Scheiner R. (2006). Downregulation of *vitellogenin* gene activity increases the gustatory responsiveness of honey bee workers (*Apis mellifera*). Behav. Brain Res. 169, 201–205. 10.1016/j.bbr.2006.01.006 16466813 PMC2398700

[B3] Ament S. A. Wang Y. Robinson G. E. (2010). Nutritional regulation of division of labor in honey bees: toward a systems biology perspective. Wiley Interdiscip. Rev. Syst. Biol. Med. 2, 566–576. 10.1002/wsbm.73 20836048

[B4] Balfanz S. Jordan N. Langenstück T. Breuer J. Bergmeier V. Baumann A. (2014). Molecular, pharmacological, and signaling properties of octopamine receptors from honeybee (*Apis mellifera*) brain. J. Neurochem. 129, 284–296. 10.1111/jnc.12619 24266860

[B5] Behrends A. Scheiner R. (2010). Learning at old age: a study on winter bees. Front. Behav. Neurosci. 4:15. 10.3389/fnbeh.2010.00015 20428511 PMC2859878

[B6] Behrends A. Scheiner R. (2012). Octopamine improves learning in newly emerged bees but not in old foragers. J. Exp. Biol. 215, 1076–1083. 10.1242/jeb.063297 22399652

[B7] Behrends A. Scheiner R. Baker N. Amdam G. (2007). Cognitive aging is linked to social role in honey bees (*Apis mellifera*). Exp. Gerontol. 42, 1146–1153. 10.1016/j.exger.2007.09.003 17976939 PMC2408866

[B8] Blenau W. Balfanz S. Baumann A. (2000). *Amtyr1*: characterization of a gene from honeybee (*Apis mellifera*) brain encoding a functional tyramine receptor. J. Neurochem. 74, 900–908. 10.1046/j.1471-4159.2000.0740900.x 10693920

[B9] Erber J. Hoorman J. Scheiner R. (2006). Phototactic behaviour correlates with gustatory responsiveness in honey bees (*Apis mellifera* L.). Behav. Brain Res. 174, 174–180. 10.1016/j.bbr.2006.07.023 16934881

[B10] Grohmann L. Blenau W. Erber J. Ebert P. R. Strünker T. Baumann A. (2003). Molecular and functional characterization of an octopamine receptor from honeybee (*Apis mellifera*) brain. J. Neurochem. 86, 725–735. 10.1046/j.1471-4159.2003.01876.x 12859685

[B11] Hunt G. J. Amdam G. V. Schlipalius D. Emore C. Sardesai N. Williams C. E.. (2007). Behavioral genomics of honeybee foraging and nest defense. Naturwissenschaften 94, 247–267. 10.1007/s00114-006-0183-1 17171388 PMC1829419

[B12] Johnson B. R. (2010). Division of labor in honeybees: form, function, and proximate mechanisms. Behav. Ecol. Sociobiol. 64, 305–316. 10.1007/s00265-009-0874-7 20119486 PMC2810364

[B13] Nilsen K.-A. Ihle K. E. Frederick K. Fondrk M. K. Smedal B. Hartfelder K.. (2011). Insulin-like peptide genes in honey bee fat body respond differently to manipulation of social behavioral physiology. J. Exp. Biol. 214, 1488–1497. 10.1242/jeb.050393 21490257 PMC3076075

[B14] Reim T. Balfanz S. Baumann A. Blenau W. Thamm M. Scheiner R. (2017). AmTAR2: functional characterization of a honeybee tyramine receptor stimulating adenylyl cyclase activity. Insect Biochem. Mol. Biol. 80, 91–100. 10.1016/j.ibmb.2016.12.004 27939988

[B15] Reim T. Scheiner R. (2014). Division of labour in honey bees: age- and task-related changes in the expression of octopamine receptor genes. Insect Mol. Biol. 23, 833–841. 10.1111/imb.12130 25187440

[B16] Reim T. Thamm M. Rolke D. Blenau W. Scheiner R. (2013). Suitability of three common reference genes for quantitative real-time PCR in honey bees. Apidologie 44, 342–350. 10.1007/s13592-012-0184-3

[B18] Roeder T. (2005). Tyramine and octopamine: ruling behavior and metabolism. Annu. Rev. Entomol. 50, 447–477. 10.1146/annurev.ento.50.071803.130404 15355245

[B19] Scheiner R. (2004). Responsiveness to sucrose and habituation of the proboscis extension response in honey bees. J. Comp. Physiol. 190, 727–733. 10.1007/s00359-004-0531-6 15185117

[B20] Scheiner R. Abramson C. I. Brodschneider R. Crailsheim K. Farina W. M. Fuchs S. (2013). “Standard methods for behavioral studies of *Apis mellifera*,” in The COLOSS BEEBOOK: Standard Methodologies for *Apis mellifera* Research. Journal of Apicultural Research (Vol. 52), eds Dietemann V. Ellis J. D. Neumann P., 1–58. 10.3896/IBRA.1.52.4.04

[B21] Scheiner R. Amdam G. V. (2009). Impaired tactile learning is related to social role in honey bees. J. Exp. Biol. 212, 994–1002. 10.1242/jeb.021188 19282496 PMC2726859

[B22] Scheiner R. Arnold G. (2010). Effects of patriline on gustatory responsiveness and olfactory learning in honey bees. Apidologie 41, 29–37. 10.1051/apido/2009040

[B23] Scheiner R. Barnert M. Erber J. (2003). Variation in water and sucrose responsiveness during the foraging season affects proboscis extension learning in honey bees. Apidologie 34, 67–72. 10.1051/apido:2002050

[B24] Scheiner R. Blenau W. Baumann A. (2006). Aminergic control and modulation of honeybee behaviour. Curr. Neuropharmacol. 4, 259–276. 10.2174/157015906778520791 18654639 PMC2475800

[B25] Scheiner R. Erber J. Page R. E. Jr. (1999). Tactile learning and the individual evaluation of the reward in honey bees (*Apis mellifera* L.). J. Comp. Physiol. A 185, 1–10. 10.1007/s003590050360 10450609

[B26] Scheiner R. Page R. E. Jr. Erber J. (2001a). Responsiveness to sucrose affects tactile and olfactory learning in preforaging honey bees of two genetic strains. Behav. Brain Res. 120, 67–73. 10.1016/s0166-4328(00)00359-4 11173086

[B27] Scheiner R. Page R. E. Jr. Erber J. (2001b). The effects of genotype, foraging role, and sucrose perception on the tactile learning performance of honey bees (*Apis mellifera* L.). Neurobiol. Learn. Mem. 76, 138–150. 10.1006/nlme.2000.3996 11502146

[B28] Scheiner R. Page R. E. Erber J. (2004). Sucrose responsiveness and behavioral plasticity in honey bees (*Apis mellifera*). Apidologie 35, 133–142. 10.1051/apido:2004001

[B29] Scheiner R. Plückhahn S. Oney B. Blenau W. Erber J. (2002). Behavioural pharmacology of octopamine, tyramine and dopamine in honey bees. Behav. Brain Res. 136, 545–553. 10.1016/s0166-4328(02)00205-x 12429417

[B30] Scheiner R. Reim T. Søvik E. Entler B. V. Barron A. B. Thamm M. (2017). Learning, gustatory responsiveness and tyramine differences across nurse and forager honeybees. J. Exp. Biol. 220, 1443–1450. 10.1242/jeb.152496 28167800

[B31] Scheiner R. Steinbach A. Claßen G. Strudthoff N. Scholz H. (2014a). Octopamine indirectly affects proboscis extension response habituation in *Drosophila melanogaster* by controlling sucrose responsiveness. J. Insect Physiol. 69, 107–117. 10.1016/j.jinsphys.2014.03.011 24819202

[B32] Scheiner R. Toteva A. Reim T. Søvik E. Barron A. B. (2014b). Differences in the phototaxis of pollen and nectar foraging honey bees are related to their octopamine brain titers. Front. Physiol. 5:116. 10.3389/fphys.2014.00116 24734024 PMC3975121

[B33] Schulz D. J. Barron A. B. Robinson G. E. (2002). A role for octopamine in honey bee division of labor. Brain Beh. Evol. 60, 350–359. 10.1159/000067788 12563167

[B34] Schulz D. J. Huang Z. Y. Robinson G. E. (1998). Effects of colony food shortage on behavioral development in honey bees. Behav. Ecol. Sociobiol. 42, 295–303. 10.1007/s002650050442

[B35] Smith T. A. (1977). Phenethylamine and related compounds in plants. Phytochemisty 16, 9–18. 10.1016/0031-9422(77)83004-5

[B36] Søvik E. Cornish J. L. Barron A. B. (2013). Cocaine tolerance in honey bees. PLoS One 8:e64920. 10.1371/journal.pone.0064920 23741423 PMC3669089

[B37] Szczêsna T. (2006). Protein content and amino acid composition of bee-collected pollen from selected botanical origins. J. Apicult. Science 50, 81–90.

[B38] Thamm M. Scheiner R. (2014). PKG in honey bees: spatial expression, *Amfor* gene expression, sucrose responsiveness, and division of labor. J. Comp. Neurol. 522, 1786–1799. 10.1002/cne.23500 24214291

[B39] Toth A. L. Kantarovich S. Meisel A. F. Robinson G. E. (2005). Nutritional status influences socially regulated foraging ontogeny in honey bees. J. Exp. Biol. 208, 4641–4649. 10.1242/jeb.01956 16326945

[B40] Toth A. L. Robinson G. E. (2005). Worker nutrition and division of labour in honeybees. Anim. Behav. 69, 427–435. 10.1016/j.anbehav.2004.03.017

[B41] Wang Y. Brent C. S. Fennern E. Amdam G. V. (2012). Gustatory perception and fat body energy metabolism are jointly affected by vitellogenin and juvenile hormone in honey bees. PLoS Genet. 8:e1002779. 10.1371/journal.pgen.1002779 22761585 PMC3386229

